# Financial Data Mining Model Based on K-Truss Community Query Model and Artificial Intelligence

**DOI:** 10.1155/2022/9467623

**Published:** 2022-10-11

**Authors:** Zhuhua Han, Feng Li, Gong Wang

**Affiliations:** ^1^School of Bid Data Science, Hebei Finance University, Baoding, Hebei 710051, China; ^2^Applied Technology Research and Development Center Wisdom, Hebei University, Baoding, Hebei 710051, China; ^3^School of Computer and Information Engineering, Hebei Finance University, Baoding, Hebei 710051, China

## Abstract

With the continuous development of Internet technology and related industries, emerging technologies such as big data and cloud computing have gradually integrated into and influenced social life. Emerging technologies have, to a large extent, revolutionized people's way of production and life and provided a lot of convenience for people's life. With the popularity of these technologies, information and data have also begun to explode. When we usually use an image storage system to process this information, we all know that an image contains countless pixels, and these pixels are interconnected to form the entire image. In real life, communities are like these pixels. On the Internet, communities are composed of interconnected parts. Nowadays, in various fields such as image modeling, we still have some problems, such as the problem of recognition rate, and we also found many problems when studying the community structure, which attracts more and more researchers, but the research on community query problems started late and the development is still relatively slow, so designing an excellent community query algorithm is a problem we urgently need to solve. With this goal, and based on previous research results, we have conducted in-depth discussions on community query algorithms, and hope that our research results can be applied to real life.

## 1. Introduction

Since the advent of the Internet in the early 1970s, a series of emerging science and technology, such as big data and cloud computing, has emerged [1]. These technologies have greatly improved people's quality of life and facilitated people's life and production work. With the popularity of these technologies, information and data have also begun to explode. When we usually use an image storage system to process this information, we all know that an image contains countless pixels, and these pixels are interconnected to form the entire image [2]. Nowadays, researchers have made in-depth discussions and research on the problems found in the community. The problems of community surveys are part of the problems found in the community [3]. The research on this problem is late and has a long process. Based on the results of previous research, the researchers designed a variety of community structures in order to extract the characteristics of the community and other data and information. Among them, the most widely used is the K-truss system, which mainly detects the relationship between pixels through a triangle formed by pixels in the image and designs an excellent community structure based on the detection results [4]. This article is mainly aimed at the K-truss system and uses algorithms to design a data model. For the smooth progress of the research, we decided to use a directed power graph for analysis [5].

## 2. Related Work

Literature pointed out that the network signal has a very important influence on the entire research process [6]. In the K-core system, we found that the signal in the network is very stable. Only the pixels of the image will affect the recognition rate of dense subimages. Therefore, in this way, we can choose a dense subgraph with firm edges and then use the number of triangles in the K-truss system to observe the firmness. Literature shows that the signal stability in the network can be obtained by the number of triangles, so we can know that the system needs to be connected to a stable signal to design a good data model in order to improve the efficiency of the entire experimental process [7]. The literature describes the method of using functions to design the K-truss system, and the method is also introduced into the MapReduce system [5]. The literature improved the method, increased the calculation efficiency, and reduced the amount of calculation. On this basis, it explained a way to deal with large graphs. Some researchers have proposed some more excellent algorithms to improve the efficiency of processing large images, such as K truss [8]. But at the same time, the problems and disadvantages of this system are also revealed in the actual application scenarios. Literature pointed out that some scholars introduced a heuristic algorithm OLAK to make up for the shortcomings of the system. This problem needs to fix *b* key points outside the K core (not deleted because of the degree), so that *k* in the original graph core becomes the largest [9]. The literature also explained the solutions to these problems and also raised some other problems, such as in the network operation, users will affect the transmission of signals and information in the network [10]. In general, in a community structure, the fewer the users, the sparser the relationship, and the more stable the signal. But if some important users go offline or the relationship between important users breaks, it will cause damage to the community structure. For example, some Zhihu users will leave Zhihu because their questions are not answered satisfactorily [11]. This may also cause other users to leave. This will break the social structure, which will also affect other different social networks [12]. Therefore, the research on the key points of the network is particularly important, which is also conducive to our maintenance of the entire social network and social structure. This problem is extended to the K-truss version in the reference [13]. It is mentioned that in a complex network structure, the actions between users will also have a huge impact on the stability of the network. Often the more users in a social network, the closer the relationship between users, the more active the social network, and the higher the stability [14]. In the network, the departure of some key users, or the break of the relationship between some key users, will have a greater impact on the structure of the entire social network, and even “collapse” the network structure.

## 3. Research on K-Truss Community Query Model

### 3.1. K-Truss Community Query Model Design

In the previous research process, we used the K-core system to standardize the pixels in the image and stipulate that the size of each pixel must be greater than the *k* value, but the edges are not standardized. In this section, we mainly use the K-truss system and regulate the length of the sides, stipulating that the sides in the K-truss system should form *k* − 2 triangles. In the actual process, we found that the K-truss system is similar to the K-core system, but the effect is better than that of the K-core system, and the results are more accurate.

We assume that there is an edge set *B* and use Tk (*B*, *G*) to represent the K-truss system after the edge set *B* is extracted. Because the set *B* of edges is extracted, which reduces the length of other edges or even cannot meet the requirements of K truss, we make these edges a subset of the set *B*. The relationship between the two is shown in the following equation:

(1) $$\begin{array}{c} F_{i}\left(B,T_{k}\left(G\right)\right)=E_{T_{k}\left(G\right)}-E_{T_{k}\left(B,G\right)}\cup B\text{.} \end{array}$$

From this, we can lead to the problem of the maximum value in the K-truss system. In the weighted directed graph *G*, we need to extract the edges of the network to form a set *B*, and the edges in the K-truss system after obtaining *B* are reduced, so we get the largest subset of *B*.

**Figure 1:**
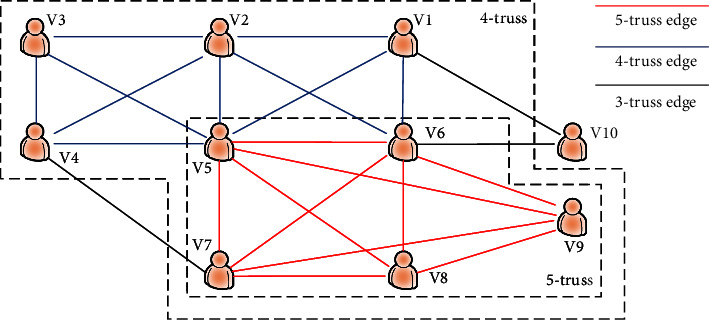
Example of K-truss minimization problem.

Figure 1 shows the process of obtaining the minimum value in the K-truss system. There is an online social platform whose users are an element of the platform, and there is an edge between every two users. At this time, the number of common concerns of the two users will reflect the familiarity of the relationship between the two. In the hypothesis process, we simulated 15 social platforms. In the K-truss system, assuming that the number of common attention of two users is greater than *k* − 2, it can indicate that the relationship between the two is not harmonious, and the number of corresponding edges will be reduced, so this will also affect the relationship with other users and even affect the entire platform. We use red, blue, and black to mark the different sides. The blue side is 5, the black side is 4, and the red side is 3.

### 3.2. Experimental Environment Design

We will explain the experimental process in detail, analyze the information base used and collected, evaluate the results, and compare the computational efficiency of various algorithms and functions to prove the accuracy of the results. The following processor is used in our experimental equipment: Intel Corei6-2450M with a memory of 9 GB. The design data model is designed using computers and monitors, and we use the cloud data platform to write algorithms. In this process, we use the *B* language.

In the research process, several algorithms are used for constructing community structure: (1) BFS Steiner tree algorithm based on social network elements, (2) Steiner tree algorithm on edges described in this article, (3) BulkDelete algorithm for collecting elements and samples, and (4) BulkDelete++ algorithm used by other researchers. In order to prove the accuracy of the results, we will compare the algorithms according to certain standards.

This article collects key data and information in the following 5 information databases to ensure the smoothness of the research. These information databases are Facebook, Amazon, Twitter, YouTube, and LiveJournal. These 5 information databases are processed through the right to have pictures.  Table 1lists the characteristics of the database, including |V| refers to the total number of users, |E| refers to the number of elements, d_max_is the meaning of on3dmax side length, and I (Ø) refers to all pixels in the truss is refers to the pictures.

Information database Twitter is a social network platform developed in the United States. This information database is obtained by collecting the information of Twitter users. This platform is composed of users and their relationships. The elements represent different users and the edges represent the relationship between the two. From the chart, we can see that the size of the element points in the Twitter information database exceeds 1085, and the *k* value in the K-truss system exceeds 89. This shows that the relationship between users is sticky and the social network is relatively mature and stable. Amazon is an online shopping platform developed in the United States. This information database is obtained through data released by Amazon. The element represents the product, and the edge represents that the product was purchased by two people. Facebook is a social networking platform developed in the United States. This information database is obtained from users and published data on Facebook. Elements represent users, and edges represent mutual friends between users. YouTube is the largest video platform in the United States. On the YouTube platform, users can follow each other in groups. The elements in the information database represent users, and the edges represent common concerns among users. LiveJournal is also an online social platform. Similar to Weibo, multiple users can form a circle of friends, which becomes an online community. The elements in the figure represent users, and the edges represent the stickiness of users in the online community.

In terms of the accuracy of the evaluation results, the evaluation index items in this study are mainly divided into two parts. The first is to judge the effect of the algorithm used in this study. By comparing the value of the original K-truss system calculated by the two algorithms, it is found that the BFS algorithm has a better performance, which proves that the algorithm used in this study has a better effect than the point-based algorithm in the original algorithm. The larger the value of *k*, the higher the accuracy of the proposed algorithm. The smaller the value of *k*, the lower the accuracy of the algorithm results. In order to make the comparison result more objective, we need to use the controlled variable method, which is to measure the time from the beginning to the end of the Steiner tree algorithm and fix the same calculation time. The calculated subgraph *G*0 uses the same parameters, but the final result of the K-truss system subgraph *G*0 is different, so we do not collect the data. Even if the key data are collected, we cannot calculate the time fixed in the same range.

The second is to compare the results of the initial BulkDelete algorithm with the improved BulkDelete++ algorithm in this article. We will compare the number of elements in the two algorithms to prove that the improved algorithm is more accurate and efficient. The two algorithms will delete the same subgraph *G*0.  Table 2 lists in detail several evaluation indicators and the number of element points in the *η* value information database, and we fixed the value of *γ* as 4, and *Q* as the number of edges. In this part, we also apply the method of controlling variables, and mainly to analyze the samples in the collection, we start with a collection of 2 samples and randomly select the following samples. In order to make the experimental process more objective, we will perform the above process 150 times to calculate the final evaluation criteria for the mean.

### 3.3. Experimental Results and Analysis

**Figure 2:**
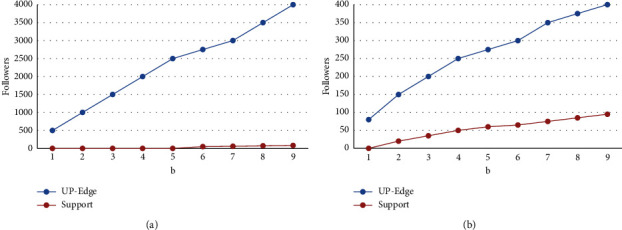
Validity experiment (change *b*). (a) Live journal (vary *b*). (b) Live journal (vary *b*).

In order to further prove the accuracy of the UP-Edge algorithm results, we also compared the subsets generated by different algorithms. Because some algorithms are very complex and computationally intensive, they may become more and more complex as the *b* set increases. Therefore, we chose two sets with fewer elements to compare the number of subsets of different algorithms. From the above figure, we can see that the number of subsets generated by the UP-Edge algorithm is similar to the number of subsets generated by the accurate algorithm, and there is basically no difference. However, the number of subsets generated by the UP-Edge algorithm is less than the number of subsets generated by the complexity algorithm. In these information databases, we have also compared the number of subsets generated by heuristic algorithms and complexity algorithms. It can be clearly seen from Figure 2 that in these selected databases, the number of generated subsets generated by the UP-Edge algorithm is less than the number of subsets generated by the complexity algorithm.

**Figure 3:**
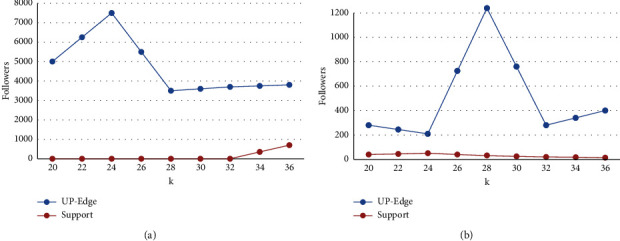
Validity experiment (change *k*). (a) Live journal (vary *k*). (b) Live journal (vary *k*).

Figure 3 shows the validity experiment, in order to prove the accuracy of the UP-Edge algorithm calculation results in different situations, and we have studied the UP-Edge algorithm and the complexity algorithm on two information databases, using different variables. In the study of variable *b*, we set *k* as 30 and *b* from 1 to 9. In the study of variable *k*, we set *b* as 6 and *k* from 21 to 30. From the figure, we can clearly see that when the value of *b* is fixed, the number of subsets of the two algorithms is proportional to the value of *k*. The smaller the value of *k*, the smaller the number of subsets. This also proves the properties mentioned in the above content and the function *f* = |Ft (*B*, Tk (*G*))| is a linear function. When more edges are deleted, the number of subsets will also increase. However, in these two information bases, the UP-Edge algorithm produces more subsets than the complexity algorithm, and the *k* value has a great influence on the generation of subsets, but it is not linear. The experimental results show that the calculation results of the UP-Edge algorithm are more accurate and efficient.

We also compared the difference between K core and K truss for selecting dense subgraphs. We compared the parameters of the two systems in the above five information databases. Before that, the specific calculation method is as follows:

(2) $$\begin{array}{c} C\left(G\right)=\frac{3\times numberoftrianglesinG}{\sum_{v\in V_{G}}\left(\begin{array}{c} de\;g\left(v\right)\\ 2 \end{array}\right)}\text{.} \end{array}$$

In order to make the comparison result more objective and credible, we have selected different elements in each image. In the K-core system, we selected 40% of the elements, and in the K-truss system, we selected the trussness of the edges and 40% of the elements. We compare the parameters of the dense submap formed by these elements. After comparison, we can see that the parameters of the dense submap selected in the truss system are all lower than those in the core system, which indicates that the result error of the truss system is smaller and easy to calculate. Moreover, the calculation complexity of the K-truss system is lower than that of the K-core system, so at the same time, the calculation speed of the K-truss system is faster.

**Figure 4:**
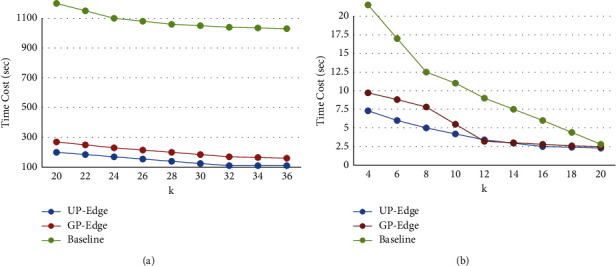
High-efficiency experiment (change *k*). (a) Live journal (vary *k*). (b) Live journal (vary *k*).

Then, the computational and operational efficiency of the UP-Edge algorithm should be evaluated. By comparing the computation time of UP-Edge algorithm, complexity algorithm, and heuristic algorithm in the same information base and the same coefficients, that is, when the coefficients of other variables are the same, the computation time of UP-Edge algorithm, complexity algorithm, and heuristic algorithm is compared. In these information databases, we set fixed parameters to ensure the objectivity of the experimental results and measured the calculation time of the three algorithms. The results show that the calculation time of the UP-Edge algorithm on all information databases is less than that of the other two algorithms, especially in the Facebook and Twitter information databases. This algorithm is 10 times faster than the complexity algorithm.  Figure 4 shows the high-efficiency experiment (change *k*).

In order to further prove the great advantages of UP-Edge, we chose two other information repositories, namely, YouTube and Amazon. We set the same parameters in these two databases for experimentation and comparison. In order to obtain the relationship between the value of *k* and the calculation efficiency of the algorithm, we set *b* = 6 in the Amazon information database and take values from 20 to 35 for *k*. In the YouTube database, we set *b* = 4 and *k* from 5 to 10. The figure shows the calculation time of the three algorithms under different *k* values. From these two datasets, we can see that the higher the value of *k*, the shorter the computation time of the algorithm. The lower the value of *k*, the longer the computation time. Since the number of edges in Tk (*G*) increases only if *k* decreases, so does the number of corresponding elements. Through these experiments, we found that the UP-Edge algorithm is optimal, with small error and high computational efficiency.

## 4. Design and Application of Financial Data Mining Model Based on Artificial Intelligence

### 4.1. Establishment of Data Mining Model

**Figure 5:**
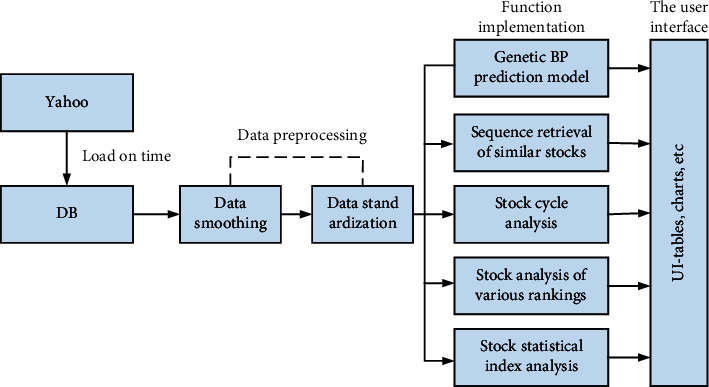
System model framework.

In this section, we will analyze the financial data processing system in the securities industry. In this system, we build a financial data estimation system on the basis of an improved genetic neural network system and also use a classification algorithm to collect key characteristics between different stocks to build a reasonable data model. The specific structure is shown in Figure 5.

In the information we collect, we refer to the data published by the global financial center or company, namely, the opening price, the high price, the low price, and the closing price. The collected data cannot be used directly, because the amount is too large and the types are too many to reduce the efficiency of the subsequent process, because we need to process, classify, and process the collected data, which can reduce the amount of calculation in the subsequent calculation process and improve efficiency.

(1) *Smoothing*. We first need to smooth the data. It is expressed as follows:
  (3) $$\begin{array}{c} {\hat{y}}_{i}=\frac{\overset{n}{\underset{i=1}{\sum }}K_{k}\left(i-t\right)y_{i}}{\overset{n}{\underset{i=1}{\sum }}K_{h}\left(i-t\right)}\text{.} \end{array}$$
Among them, the heuristic function is as follows:
  (4) $$\begin{array}{c} K_{h}\left(x\right)=\frac{1}{h\sqrt{2\pi }}e^{s^{2}/2k^{2}}\text{.} \end{array}$$
(2) *Standardization*. In the previous research, we used the sigmoid function to analyze the neural network, but this function can only identify data in the binary system, so we need to express these data in the binary system. Nowadays, many researchers have explained different functions. Through research, we have found a function with a better effect. The specific formula is as follows:
  (5) $$\begin{array}{c} {\bar{x}}_{i-1}=\frac{0.1-0.9}{\min\left(X_{i}\right)-\max\left(X_{t}\right)}x_{i-1}+\frac{0.9\;\min\left(X_{i}\right)-0.1\;\max\left(X_{t}\right)}{\min\left(X_{i}\right)-\max\left(X_{t}\right)}\text{.} \end{array}$$

After the data are expressed in the binary system, the calculation is performed, and the calculated result needs to be analyzed in the decimal system, so we need a second conversion.

During the research process, we performed operations such as calculating the average, shifting the average, and formulating evaluation standards. In the financial data analysis system, investors need to consult various information and data about stocks, including closing prices, opening prices, highest prices, and turnover. Therefore, we entered the following information into the system and processed it:

1. *Basic Information*. Opening price, closing price, highest price, lowest price, transaction amount, drop rate, etc.
2. *Change Information*. Weekly change, monthly change, quarterly change, annual change, etc.

### 4.2. System Development and Design

This system uses a three-tier architecture model, which is now the most commonly used model, mainly composed of three parts: information layer, processing layer, and display layer. The information layer is mainly used to collect different data and information and transcode them so that they can be transmitted to other platforms. This layer is to provide device support through computers, databases, and cloud platforms. The processing layer is the transitional part between the information and the display layer. It is mainly used to process information and classify and integrate data according to the parameters set by the experimenter. After processing according to certain rules, these data are then transmitted to other devices and the display layer. The display layer is mainly supported by the display as a device, and users can check and obtain information on this layer.

In this process, we mainly use Python to complete the design of the entire algorithm and use Microsoft Visual Studio 2006 to provide equipment support. This computer is fully functional and complete, runs fast, can process a variety of data at the same time, and has a large memory, these calculation results can be stored, real-time data transmission can also be realized, and the data can be transmitted to other platforms. In the research process, we can know that the database and information library play a great role in the experiment. In order to keep the data complete and long-term, we chose Microsoft's Microsoft SQL Service 2006 system, which has a large memory and can also interact with multiple devices that are connected. After that, we used PowerDesignerl0 to construct models and systems. This device can complete the construction of multiple models with high efficiency and low cost.

### 4.3. Similar Stock Retrieval Model

We have explained the relevant data model and database in detail. In this section, we analyze the stock characteristics and the market. We will use the data system to process and integrate the stocks, then collect the characteristics of the stocks, and then use the classified. The method ranks the stocks, and we also evaluate the authenticity of these data through corresponding standards to ensure the smooth progress of the following process.

First, we need to analyze the Euclidean distance. The principle is to extract variables at different times as an element in the Euclid chart, then estimate the distance between these variables according to the distance between the elements in the Euclid icon, and use these distances as a fixed standard, and the specific formula is as follows:

(6) $$\begin{array}{c} D\left(X,Y\right)={\overset{n}{\underset{i=1}{\sum }}\left(X_{i}-Y_{i}\right)}^{2}\text{.} \end{array}$$

Other scholars have also proposed another method, the principle of which is to use a linear regression function to estimate an approximate value, correspond the initial value to the parameters in the function, calculate the Euclidean distance, then use the initial time series as a set, classify the elements in this set, and get a subset:

(7) $$\begin{array}{c} f\left(t,w\right)=w_{0}+w_{1}t+w_{2}t^{2}+\cdots +w_{p-1}t^{p-1}\text{.} \end{array}$$

We divide the set into seven subsets, which include *k* elements. If an element in this set belongs to an unknown pattern, then this element is the key point of this pattern. When the coefficient *f* > 0, the element is the key point of mode P. The necessary and insufficient conditions for ￡∈*x* not to belong to the unknown mode are as follows:

(8) $$\begin{array}{c} d\left(x\prime ,x\right)<\frac{\zeta }{2}\text{.} \end{array}$$

From the perspective of big data, the sequence set is composed of randomly selected variables, so it is also a set of known and unknown elements. Known elements are composed of basic information of data, unknown elements are composed of random samples, and different data models are also included. Because linear parameters are unknown elements of the sequence set, some scholars use ACF to calculate the similarity value of the sequence set. He first collected 25 elements in the sequence set, matched these elements with ACF values one to one, and then processed and classified these data and information. The calculation formula is as follows:

(9) $$\begin{array}{c} r_{k}=\frac{\overset{n-k}{\underset{i=1}{\sum }}\left(y_{i}-{\bar{y}}_{i}\right)\left(y_{i+k}-{\bar{y}}_{i+k}\right)}{\sqrt{\overset{n-k}{\underset{i=1}{\sum }}{\left(y_{i}-{\bar{y}}_{i}\right)}^{2}\overset{n-k}{\underset{i=1}{\sum }}{\left(y_{i+k}-{\bar{y}}_{i+k}\right)}^{2}}}\text{.} \end{array}$$

Among them,

(10) $$\begin{array}{c} r{\bar{y}}_{t}=\frac{1}{n-k}\overset{n-k}{\underset{t=1}{\sum }}y_{t}\text{,}{\bar{y}}_{t+k}=\frac{1}{n-k}\overset{n-k}{\underset{t=1}{\sum }}y_{t+k}\text{.} \end{array}$$

It is connected with other data systems and has corresponding values. Therefore, we can see that it is also a linear function:

(11) $$\begin{array}{c} x_{t}=a_{1}x_{t-1}+a_{2}x_{t-2}+\cdots +a_{p}x_{t-p}+\varepsilon_{t}\text{.} \end{array}$$

The coefficients in the PR data system can be judged from the following formula:

(12) $$\begin{array}{c} c_{n}=\left\{\begin{array}{cc} -\alpha_{1}\text{,} & ifn=1\\ -\alpha_{n}-\overset{p}{\underset{m=1}{\sum }}\left(1-\frac{m}{n}\right)\alpha_{m}c_{n-m}\text{,} & if1<n\leq p\text{,}\\ -\overset{p}{\underset{m=1}{\sum }}\left(1-\frac{m}{n}\right)\alpha_{m}c_{n-m}, & ifp<n\text{.} \end{array}\right. \end{array}$$

The collected coefficients basically meet the requirements of the sequence collection, so it is sufficient to collect the coefficients of the previous part. These coefficients are relatively stable and will not change with changes in experimental data. Among them, the Euclidean distance can be used to evaluate the similarity between sequence sets, which can simplify the calculation process and improve the calculation efficiency. It is very suitable for processing complex data of different types.

The FCM algorithm is used to classify and integrate data, and its main principle is to select a subset of *X* × *Y* fuzzy. Fuzzy relations need to be converted into binary, and the specific process is as follows:

(13) $$\begin{array}{c} R:X\times Y⟶\left[0,1\right]\text{,}\\ \left(x,y\right)\mapsto R\left(x,y\right)\text{.} \end{array}$$

The principle of FCM is mainly to correspond the minimum value of the linear function *L* to the set *X* to obtain an approximate value and then perform data classification. The calculation formula of the linear function is as follows:

(14) $$\begin{array}{c} MinimizeJ_{m}\left(X:U,V\right)={\overset{c}{\underset{i=1}{\sum }}\overset{n}{\underset{j=1}{\sum }}{\left(u_{ij}\right)}^{m}\left\|x_{j}-v_{i}\right\|}^{2}\text{.} \end{array}$$

Later, we discussed the results of the previous formula by classification:

(15) $$\begin{array}{c} u_{ij}\left\{\begin{array}{cc} {\left[\overset{c}{\underset{r=1}{\sum }}{\left(\frac{\left\|x_{j}-v_{i}^{0}\right\|}{\left\|x_{j}-v_{r}^{0}\right\|}\right)}^{\frac{2}{m-1}}\right]}^{-1}\text{,} & if\left\|x_{j}-v_{i}^{0}\right\|≻0\left(1\leq j\leq n,1\leq i\leq c\right)\text{,}\\ 1\text{,} & if\left\|x_{j}-v_{i}^{0}\right\|=0\text{,}\\ 0\text{,} & 0if\exists r,r\neq i,\left\|x_{j}-v_{i}^{0}\right\|=0^{\prime }\text{,} \end{array}\right.\\ v_{i}=\frac{\overset{n}{\underset{j=1}{\sum }}{\left(u_{ij}\right)}^{m}x_{j}}{\overset{n}{\underset{j=1}{\sum }}{\left(u_{ij}\right)}^{m}},1\leq i\leq c\text{.} \end{array}$$

Considering the aforementioned AR algorithm, we can formulate a series of standards to evaluate the parameters, and the process is as follows:

(16) $$\begin{array}{c} V_{xie}\left(U,V,c\right)=\frac{\overset{n}{\underset{k=1}{\sum }}\overset{c}{\underset{i=1}{\sum }}u_{ki}^{m}{\left\|v_{i}-x_{h}\right\|}^{2}}{n\times \min_{i\neq j}{\left\|v_{i}-x_{j}\right\|}^{2}}\text{.} \end{array}$$

The *V* value is the ratio of the calculation time of the function to the size of the dataset, and the *U* value can measure the number of samples in the set. The algorithm of *V* value proposed by S. H. Kwon is as follows:

(17) $$\begin{array}{c} V_{hren}\left(U,V,c\right)=\frac{\overset{c}{\underset{i=1}{\sum }}\overset{n}{\underset{j=1}{\sum }}u_{ki}^{m}{\left\|v_{j}-x_{i}\right\|}^{2}+1/c\overset{c}{\underset{i=1}{\sum }}{\left\|v_{i}-\right\|}^{2}}{\min_{i\neq j}{\left\|v_{i}-x_{j}\right\|}^{2}}\text{.} \end{array}$$

This function improves calculation efficiency. When the value of *C* becomes smaller, it will be farther and farther away from the value of *n*, and the final result will be greater than 0. At this time, there will be an error in the result. So, S. H. Kwon proposed the concept of a penalty function to reduce the error and improve the accuracy of the result. At the same time, H. Sun-S. and Q. Jiang also proposed the algorithm of *V* value:

(18) $$\begin{array}{c} V_{WSJ}\left(U,V,c\right)=Scat\left(c\right)+\frac{Sep\left(c\right)}{Sep\left(C_{\max}\right)}\text{.} \end{array}$$

Among them,

(19) $$\begin{array}{c} Scat\left(c\right)=\frac{1/c\overset{c}{\underset{j==1}{\sum }}\left\|\sigma \left(v_{i}\right)\right\|}{\left\|\sigma \left(X\right)\right\|}\text{,}\\ Sep\left(c\right)=\frac{D_{\max}^{2}}{D_{\min}^{2}}\overset{c}{\underset{i=1}{\sum }}{\left({\overset{c}{\underset{j=1}{\sum }}\left\|v_{i}-v_{j}\right\|}^{2}\right)}^{-1}\text{.} \end{array}$$

Scat (*c*) represents the accuracy of the result, and its principle is to reduce the error of the result. Later, Dae Won Kim also proposed the calculation method of *V* value:

(20) $$\begin{array}{c} V_{OS}\left(c,U\right)=\frac{Overlap^{N}\left(c,U\right)}{Sep^{N}\left(c,U\right)} \end{array}$$

Among them,

(21) $$\begin{array}{c} Overlap^{N}\left(c,U\right)=\frac{Overlap\left(c,U\right)}{Overlap_{\max}}\text{,}\\ Overlap\left(c,U\right)=\frac{1}{c\left(c-1\right)}\overset{c-1}{\underset{p=1}{\sum }}\overset{c}{\underset{q=p+1}{\sum }}\times \left[\underset{n}{\sum }\overset{n}{\underset{j=1}{\sum }}\delta \left(x_{j},u:\right)\omega \left(x_{j}\right)\right]\;\\ \delta \left(x_{j},u:{\bar{F}}_{p},{\bar{F}}_{q}\right)=\left\{\begin{array}{c} \omega \left(x_{j}\right),if\left(\mu_{{\bar{F}}_{p}}\left(x_{j}\right)\geq u\right)an\;d\left(\mu_{{\bar{F}}_{q}}\left(x_{j}\right)<u\right)\text{,}\\ 0.0,otherwise\text{,} \end{array}\right.\\ Sep^{N}\left(c,U\right)=\frac{Sep\left(c,U\right)}{Sep_{\max}}\text{,}\\ Sep\left(c,U\right)=1-\min_{p\neq q}\left[\max_{x\in X}\min\;\left(\mu_{{\bar{F}}_{p}}\left(x\right),\mu_{{\bar{F}}_{q}}\left(x\right)\right)\right]\text{.} \end{array}$$

After many experiments, we found that some of the algorithm results have errors, or the original data has been destroyed, so we have improved them, and we will introduce them in detail later.

Nowadays, many standards are related to “compactness” and “separation.” Compactness refers to the situation where data are very tightly distributed, and separation refers to the situation where data distribution is very sparse. Effectiveness is to find the maximum compactness and minimum separation. In the process of research, we found that the previous effectiveness index has some drawbacks, and we have improved it. The calculation formula is as follows:

(22) $$\begin{array}{c} \sigma \left(x_{j},u,C_{p},C_{q}\right)=\left\{\begin{array}{c} \varepsilon_{1}^{1/m}0.65≺u^{\prime }\leq 1.0\text{,}\\ \varepsilon_{1}^{1/m}0.55\leq u^{\prime }\leq 0.65\text{,}\\ \varepsilon_{1}^{1/m}0.50\leq u^{\prime }≺0.55\text{.} \end{array}\right. \end{array}$$

At this time, we use the linear regression function to calculate the *C* value, which has a great influence on the final result. When the *c* value is obtained, the following calculations are performed:

(23) $$\begin{array}{c} Sup\left(U,V,c\right)\prime =\frac{2}{c\left(c-1\right)}\overset{c-1}{\underset{p=1}{\sum }}\overset{c}{\underset{q=p-1}{\sum }}\lambda \left(u,C_{p},C_{q}\right),\\ =\frac{2}{c\left(c-1\right)}\overset{c-1}{\underset{p=1}{\sum }}\overset{c}{\underset{q=p-1}{\sum }}\overset{n}{\underset{i=1}{\sum }}\sigma \left(x_{i},u,C_{p},C_{q}\right)\text{.} \end{array}$$

Then, we proceed to the following process:

(24) $$\begin{array}{c} Sup\left(U,V,c\right)=\frac{Sup\left(U,V,c\right)\prime }{\max_{e}Sup\left(U,V,c\right)\prime }\text{.} \end{array}$$

### 4.4. System Function Design and Realization

The composition and structure of the system are explained in detail, including some data processing modules. The module structure of the system is mainly composed of data processing and prediction, access analysis, classification, and integration. The first is data processing and prediction. In this module, the main functions are data collection and processing. First, we must collect the data released by the financial center, update the data in real time every day, classify and integrate the data, and put them into different sets in order. We design a system with two independent modules, which is a kind of intelligent system for stock prediction based on the genetic BP neural network model. The system modules are the initial genetic BP data prediction system and the improved genetic BP data prediction system. By comparing the results of the two prediction systems, the superiority of the improved system can be confirmed. In the reference analysis module, we use ACF to collect the characteristics of stocks. Later, in similar data, we will enter these data and do not consider the time factor, which greatly improves efficiency. Below, we will conduct experiments on the KDJ and RSI indicators. The first is the KDJ indicator. The steps involved in the calculation process are as follows:

(1) First, we calculate the RSV value in a period, and here, we calculated it in nine days:
  (25) $$\begin{array}{c} nΗRSV=\left(C_{m}-L_{m}\right)+\left(H_{n}-L_{n}\right)\times 100\text{.} \end{array}$$
The symbols in the formula and their meanings are as follows: Cn represents the opening price on day *n* and *L*_*n*_ represents the highest price on day *N*. After calculating the RSV value, the value shows that the fluctuation is between 1 and 150.
(2) Then, we calculate the *K* and *D* values as follows:
*K* value of the day = 1/3 × RSV of the day + 2/3 × *K* value of the day before
*D* value of the current day = 1/3 × *K* value of the current day + 2/3 × *D* value of the previous day
(3) Then, we calculate the *J* value, *J* = 3*D* − 2*K*.
After calculating the RSI indicator, it is also necessary to choose the period. We have selected the three periods of 5, 10, and 15 days. The value of the RSI indicator fluctuates from 0–150. If it exceeds 75, it is a strong market, and if it does not exceed 50, it is a weak market.

### 4.5. Experiment and Result Analysis

After that, we conducted a lot of experiments. In the experiment, we set *m*  =  2, *C*min = 2, and *C*max = 10. The specific operation process is as follows:

First, we must test the sample set. The sample set consists of 35 variables. We divide it into 3 categories for comparison experiments. The results are listed in Table 3.

Then, we tested the second set of sample set IRIS and also divided the samples into 3 categories for controlled variable tests. The results are listed in Table 4.

After that, we will test the third sample set, which includes 6 types of two-dimensional data. Through the linear regression function, we can see the results, as listed in Table 5.

Finally, we conduct experiments on the fourth sample set, and the results are shown in Table 6.

Through these experiments, we can see that the improved indicators make up for the previous deficiencies, improve efficiency, and reduce costs and errors.

## 5. Conclusion

Since the emergence of the Internet in the middle and late 20th century, emerging technologies such as big data and cloud computing have also emerged. These technologies, to a great extent, have revolutionized people's way of production and life, not only accelerating the efficiency of production, but also facilitating people's life. The proliferation of technology has also led to an exponential explosion of information and data. Especially in recent years, with the rapid development of emerging technologies, especially the remarkable development of the Internet and the spread and rise of network social platforms, many researchers begin to pay attention to the related research in this field. In this case, we chose two subgraph systems, namely, K-core system and K-truss system, and compared the effects of the two systems. Based on the K-core and K-truss systems, we found two problems, namely, the K-core maximization problem and the K-truss minimization problem. We collect the edges in the social network structure by solving these two problems. Because this problem is too complicated and takes a long time, we have chosen a heuristic algorithm to solve this problem in order to simplify the calculation process and improve calculation efficiency. We also designed related models to optimize the structure of the social network platform, hoping that our research results can solve real-life problems.

## Figures and Tables

**Table 1:** Real dataset statistics.

Dataset	(*V*)	(*E*)	dmax	*i* (∅)
Facebook	4 K	88 K	1045	80
Amazon	335 K	926 K	549	7
DBLP	317 K	1 M	342	86
YouTube	1.1 M	3 M	28754	19
LiveJournal	4 M	35 M	14815	253

**Table 2:** Experimental parameters.

Parameter	Description
*γ*	The penalty coefficient in the truss distance formula
*Q*	Query node collection
*η*	Threshold to control the size of the expanded subgraph

**Table 3:** X30 (class 3) experimental results.

Index	*V *_xte_	*V *_baid_	*V *_kwon_	*V *_HR_	*V *_vmj_	*V *_OB_	*V *_xij_
Result	3	10	2	3	3	3	3

**Table 4:** IRIS (class 3) experimental results.

Index	*V *_xte_	*V *_baid_	*V *_kwon_	*V *_HR_	*V *_vmj_	*V *_OB_	*V *_xij_
Result	2	10	2	2	3	2	3

**Table 5:** Dataset three (6 categories) test results.

Index	*V *_xte_	*V *_baid_	*V *_kwon_	*V *_HR_	*V *_vmj_	*V *_OB_	*V *_xij_
Result	6	10	6	3	6	6–10	6

**Table 6:** Experimental results of dataset 4.

Index	*V *_xte_	*V *_baid_	*V *_kwon_	*V *_HR_	*V *_vmj_	*V *_OB_	*V *_xij_
Result	4	10	6	4	5	4	5

## Data Availability

The data used to support the findings of this study can be obtained from the corresponding author upon request.
